# Viral-Based Gene Editing System for Nutritional Improvement of Fructan Content in Lettuce

**DOI:** 10.3390/ijms26062594

**Published:** 2025-03-13

**Authors:** Yarin Livneh, Dor Agmon, Ehud Leor-Librach, Alexander Vainstein

**Affiliations:** Institute of Plant Sciences and Genetics in Agriculture, The Robert H. Smith Faculty of Agriculture, Food and Environment, The Hebrew University of Jerusalem, Rehovot 76100001, Israel; yarin.livneh@mail.huji.ac.il (Y.L.);

**Keywords:** CRISPR, gene editing, biofortification, fructan, lettuce, TRV, inulin

## Abstract

Lettuce is a globally cultivated and consumed leafy crop. Here we developed an efficient tobacco rattle virus (TRV)-based guide RNA (gRNA) delivery system for CRISPR/Cas editing in the commercial lettuce cultivar ‘Noga’. Plants stably expressing Cas9 were inoculated with TRV vectors carrying gRNAs targeting five nutrient-associated genes. The system achieved an average editing efficiency of 48.7%, with up to 78.9% of regenerated plantlets showing independent mutations. This approach eliminates the need for antibiotic selection, simplifying tissue culture processes. The system supports diverse applications, including Cas12a editing and large-fragment deletions using dual gRNA sets. Targeting the *fructan 1-exohydrolase 2* (*1-FEH2*) gene produced knockout lines with significant increases in prebiotic dietary fibre fructan content, up to 5.2-fold, and an average rise in the degree of polymerisation by 2.15 units compared with controls. Combining *1-FEH1* and *1-FEH2* knockouts did not further increase fructan levels, revealing *1-FEH2* as the predominant isozyme in lettuce. RT-qPCR analysis showed reduced expression of the upstream biosynthetic enzyme *sucrose:sucrose 1-fructosyl transferase* (*1-SST*), suggesting potential feedback inhibition in fructan metabolism. This TRV-based gene editing approach, utilised here to increase fructan content, could be applied to improve other valuable traits in lettuce, and may inspire similar systems to enhance nutritional content of crops.

## 1. Introduction

Environmental challenges due to climate change, together with growing world population, demand rapid adaptability in crop improvement [1]. In the past decade, CRISPR/Cas systems emerged as invaluable tools for advancing crop production, resilience, and quality [2]. Enhancing the nutritional quality of crops is particularly important for promoting health benefits and addressing micronutrient deficiencies prevalent in more than 20% of the global population [3,4]. For example, dietary fibre is essential for health, yet intake is insufficient globally, highlighting the need for enhanced fibre-rich foods to help bridge this “fibre gap” [5,6].

The CRISPR/Cas9 system is currently the leading and most widely used technique for genome editing [7]. This system employs a single-guide RNA (gRNA) to direct the Cas9 nuclease to specific genomic sites, where it induces a double-strand break (DSB) in the DNA. Further developments of this methodology, such as base and prime editing, also rely on guiding RNA sequences to direct the enzymes to their targets and in some cases provide a template for induced genetic changes, offering a versatile molecular toolbox for precise genome modifications [8].

A main challenge in utilising CRISPR/Cas technology is the effective delivery of reagents into plant cells [2]. *Agrobacterium*-mediated transformation is the most commonly used and reliable method, but its efficiency is variable, with transgene expression often being unpredictable or inconsistent due to variability linked to the integration site [9]. Additionally, *Agrobacterium* infection is localised, which reduces the likelihood of affecting regenerative tissue, and the latter necessitates complex selection and regeneration protocols [10]. To address these limitations, viral vector systems have been developed to deliver the gRNA to plants that already stably express Cas9 [11,12]. For instance, tobacco rattle virus (TRV) is a positive-sense single-stranded RNA virus, belonging to the genus *Tobravirus*. Its compact genome enables systemic movement and replication without host genome integration. TRV consists of two RNA molecules: RNA1 encodes replication and movement proteins, while RNA2 encodes the coat protein and is used for the insertion of foreign sequences. cDNA clones of both RNA1 and RNA2 are commonly placed under 35S promoters in T-DNA vectors (pTRV1 and pTRV2) to facilitate efficient expression in plant cells [13]. This system allows for the delivery of heterologous sequences, including gRNAs for CRISPR-based editing, with high efficiency and broad tissue targeting [12]. TRV-mediated gRNA delivery has been successfully demonstrated in model plants such as *Nicotiana benthamiana* [14] and more recently in petunia [15]. With a host range of over 400 species across 50 plant families [16], TRV holds significant potential for broader application in various plant species, enhancing the scope of efficient CRISPR/Cas-based gene editing.

Lettuce (*Lactuca sativa* L.) is an important crop in the Asteraceae family, cultivated and consumed worldwide; yearly global production exceeds 27 million tonnes [17] (data include chicory). The most commonly grown lettuce types are iceberg, romaine (cos), butterhead, and loose-leaf varieties, which can be cultivated in diverse agricultural systems, such as open-field, greenhouse, and hydroponic methods [18]. With its low caloric value and appealing texture and taste, lettuce is a popular, healthy choice for salads and sandwiches. However, it is relatively low in nutrients, including dietary fibre, compared to other leafy vegetables such as kale, spinach, and chard [19,20,21,22]. Similar to other Asteraceae members, dietary fibre in lettuce includes inulin-type fructans, which are water-soluble reserve carbohydrates composed of at least two fructose (F) units, with or without a terminal glucose (G) molecule, connected through β(2→1) linkages [23,24].

Fructan metabolism is carried out by three key enzymes: Sucrose:sucrose 1-fructosyltransferase (1-SST) initiates the synthesis of 1-kestose (GFF) in the vacuole by transferring a fructose unit from sucrose (GF) to another sucrose molecule. Fructan:fructan 1-fructosyltransferase (1-FFT) then extends the chain by adding fructose units from 1-kestose or longer fructan donors, creating molecules (GF_n_) of a higher degree of polymerisation (DP). When the plant is in need of using these carbohydrate reserves, fructan 1-exohydrolase (1-FEH) catalyses the degradation of fructans by hydrolysing terminal fructose units (GF_n_ → GF_n−1_ + F) [23,25]. The main fructan types identified in lettuce are typically short (DP 3-5) [26,27,28]. Consumption of short-chain inulin-type fructans (DP < 10), also known as fructooligosaccharides (FOS), has been associated with various health benefits, such as stimulating beneficial gut microbiota, enhancing immune function, reducing digestive disorders and colon cancer risk, improving mineral absorption, and lowering obesity-related disorders [29,30,31,32,33,34].

Extensive genotyping of chicory cultivars revealed that a loss-of-function mutation in the *1-FEH2b* gene was statistically associated with reduced susceptibility to post-harvest inulin depolymerisation [35]. In lettuce, overexpression of bacterial *asparagine synthetase A* (*asnA*) resulted in 3.3-fold and 6.9-fold increases in GF_2_ and GF_4_ fructans, respectively [36]. A follow-up study on these transgenic lines indicated that fructan accumulation was linked to the upregulation of the biosynthetic enzymes 1-SST and 1-FFT, along with a marked repression of 1-FEH2 activity [37]. These studies position 1-FEH as a promising target for fructan enhancement in crops.

In this study, we established an efficient TRV-mediated gene editing system and employed it to enhance fructan content in the commercial lettuce cultivar ‘Noga’. To evaluate the efficiency of the system, we targeted five distinct genes associated with the production of key nutrients, including ascorbic acid, carotenoids, and fructans, achieving an average editing efficiency of 35.6%. Detailed analysis of four *1-FEH2* independent knockout lines revealed a significant increase in fructan content, with up to a 5.2-fold rise. This increase was accompanied by an average rise of 2.3 units in mean DP. Our findings highlight the potential of viral-based gene editing in overcoming major bottlenecks in the delivery of editing components for the purpose of biofortification and the enhancement of other valuable crop traits.

## 2. Results

### 2.1. Establishment of Efficient Virus-Mediated Gene Editing System in Lettuce

To optimise regeneration in the lettuce cultivar ‘Noga’, cotyledon explants were cultivated on media containing varying concentrations of 6-benzylaminopurine (BA) and 1-naphthaleneacetic acid (NAA) for several weeks, evaluating each composition for callus viability and plantlet regeneration (Figure S1, Table S1). Regeneration Medium 6, containing BA and NAA at a 12.5:1 ratio (0.5 mg/L and 0.04 mg/L, respectively), yielded the highest average regeneration rate of 87.3%, calculated as the number of regenerated plantlets from a number of initial explants, and was selected for all subsequent experiments with the cv. ‘Noga’.

**Figure 1:**
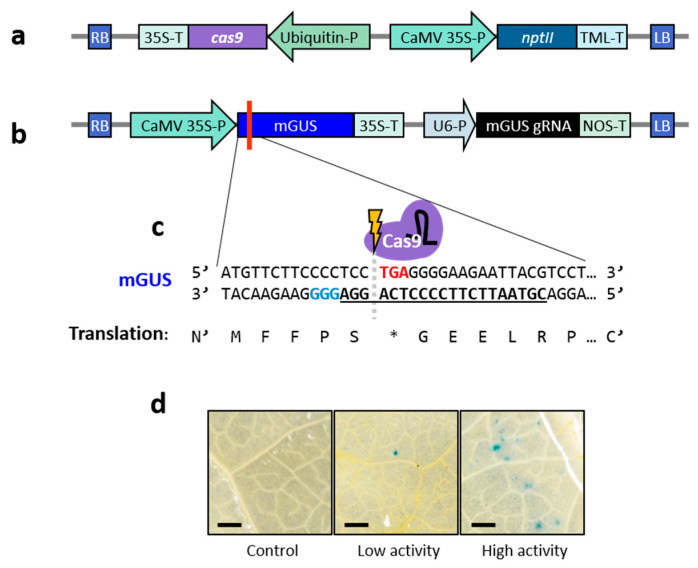
Generation of lettuce cv. ‘Noga’ plants expressing Cas9. (**a**) Schematic representation of the T-DNA region of the binary plasmid pCGN carrying UbqP:hCas9 (pCGN-UbqP:hCas9), used for generation of Cas9-expressing lettuce plants. RB = right border; LB = left border; *cas9* = human codon-optimised Cas9 gene; 35S-P/35S-T = cauliflower mosaic virus (CaMV) 35S promoter/terminator; Ubiquitin-P = *Arabidopsis thaliana* Ubiquitin 10 promoter; *nptII* = neomycin phosphotransferase II gene; and TML-T = *A. tumefaciens* tumour morphology large (TML) gene terminator. (**b**) Schematic representation of the T-DNA region of the binary plasmid pRCS carrying the mutated β-glucuronidase (mGUS) gene and its gRNA (pRCS-mGUS/gRNA). RB = right border; LB = left border; 35S-P/35S-T = CaMV 35S promoter/terminator; U6-P = U6 small nuclear RNA promoter; and NOS-T = nopaline synthase terminator. The red line indicates the location of the premature stop codon in the mGUS gene. (**c**) Nucleotide and amino acid sequences of the 5′ region of the mGUS gene. The premature stop codon is shown in red and translated as “*”; the gRNA spacer sequence is underlined and bold; the protospacer adjacent motif (PAM) sequence is highlighted in blue. (**d**) GUS histochemical staining of four-month-old control and Cas9-expressing ‘Noga’ lettuce leaves Agro-infiltrated with the pRCS-mGUS/gRNA construct. Blue spots indicate restoration of GUS activity due to Cas9-mediated gene editing. Panels display leaf surfaces from wild-type ‘Noga’ leaves (control) and transgenic plants exhibiting low- or high-Cas9-activity (few or many) blue spots. Bar = 0.5 mm.

To generate stably expressing Cas9 lettuce plants, ‘Noga’ cotyledons were transformed using *Agrobacterium tumefaciens* AGL0 carrying a binary pCGN vector. This vector contained human codon-optimised Cas9 driven by the *Arabidopsis thaliana* ubiquitin 10 promoter and the neomycin phosphotransferase II (*nptII*) gene under the CaMV 35S promoter for selection *in planta* (pCGN-UbqP:hCas9; Figure 1a). The presence of Cas9 in regenerated plants was confirmed via PCR (Figure S2). To validate Cas9 activity, leaves of four-month-old greenhouse-grown T₀ plants were infiltrated with *A. tumefaciens* carrying a pRCS vector harbouring a mutated *uidA* gene encoding β-glucuronidase (GUS) with a stop codon 12 bp downstream of the start codon (mGUS) under the CaMV 35S promoter, and a Cas9-compatible gRNA under the U6 promoter targeting this stop codon (pRCS-mGUS/gRNA; Figure 1b,c) [15,38]. Deletion of the stop codon, guided by the gRNA, could restore GUS activity if the original reading frame was maintained, providing a visual indicator of active Cas9 in the infiltrated tissue. Histochemical staining revealed blue spots at infiltration sites, signifying GUS restoration, with the frequency of spots used to classify plants as showing high or low Cas9 activity (Figure 1d). No spots were observed in wild-type ‘Noga’ control leaves infiltrated with the same vector. Plants showing high Cas9 activity were self-pollinated to produce T₁ seeds used in subsequent experiments.

**Figure 2:**
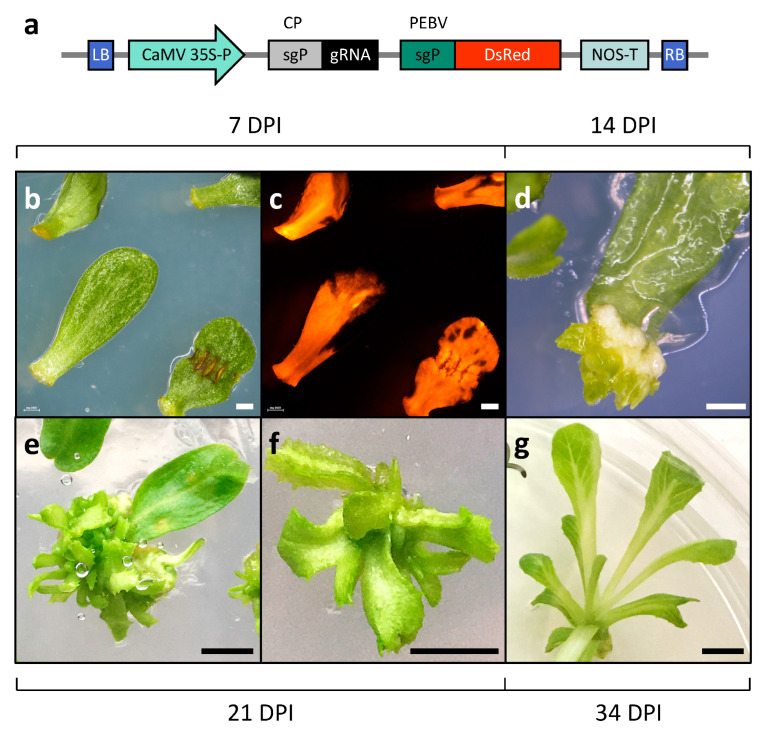
Regeneration of gene-edited lettuce cv. ‘Noga’ plants following TRV-mediated gRNA delivery. (**a**) Schematic representation of the T-DNA region of the vector used for TRV-mediated gRNA delivery to Cas9-expressing plants (pTRV2-gRNA-DsRed). LB = left border; 35S-P = cauliflower mosaic virus (CaMV) 35S promoter; sgP = sub-genomic promoter of TRV coat protein (CP); DsRed = *Discosoma* red fluorescent protein; gRNA = single-guide RNA (scaffold + spacer); PEBV = pea early browning virus; NOS-T = nopaline synthase terminator; and RB = right border. The DsRed gene serves as a marker for TRV expression. The gRNA spacer sequence was designed to target specific genes of interest. (**b**,**c**) Cotyledon explants were inoculated with the virus, and images were taken seven days post-inoculation (DPI = days post-inoculation). (**b**) Bright-field image; (**c**) fluorescence under DsRed filter. (**d**,**e**) Shoot regeneration from cotyledons at 14 DPI and 21 DPI, respectively. (**f**,**g**) Regenerated plantlet at 21 DPI and 34 DPI, respectively. Bar in (**b**–**d**) = 0.1 cm; bar in (**e**–**g**) = 0.5 cm.

To evaluate gene editing efficiency in lettuce via TRV-mediated gRNA delivery, five endogenous genes associated with nutrient production or accumulation were targeted in Cas9-expressing plants: *1-FEH2* (fructans) [39], *LCY-ε* and *CCD4a* (carotenoids) [40,41], and *GGP2* and *CSN5* (ascorbic acid) [42,43]. Specific gRNAs were designed using CRISPOR to target a restriction enzyme recognition site, enabling mutation detection via digestion disruption [44]. Each gRNA was cloned into a pTRV2 vector under TRV’s coat protein subgenomic promoter, alongside a *DsRed* marker gene under the PEBV subgenomic promoter (pTRV2-gRNA-DsRed; Figure 2a) [11]. To target a specific gene, cotyledons from ‘Noga’ Cas9-expressing plants were co-inoculated with pTRV1 mixed with one of the five pTRV2-gRNA-DsRed constructs. Cotyledons displayed extensive red fluorescence in over 90% of the explants by four to six days post-inoculation (DPI), indicating effective viral spread and expression throughout the explant (Figure 2b,c). Shoot organogenesis and plantlet formation were typically observed within 19–23 DPI (Figure 2).

**Figure 3:**
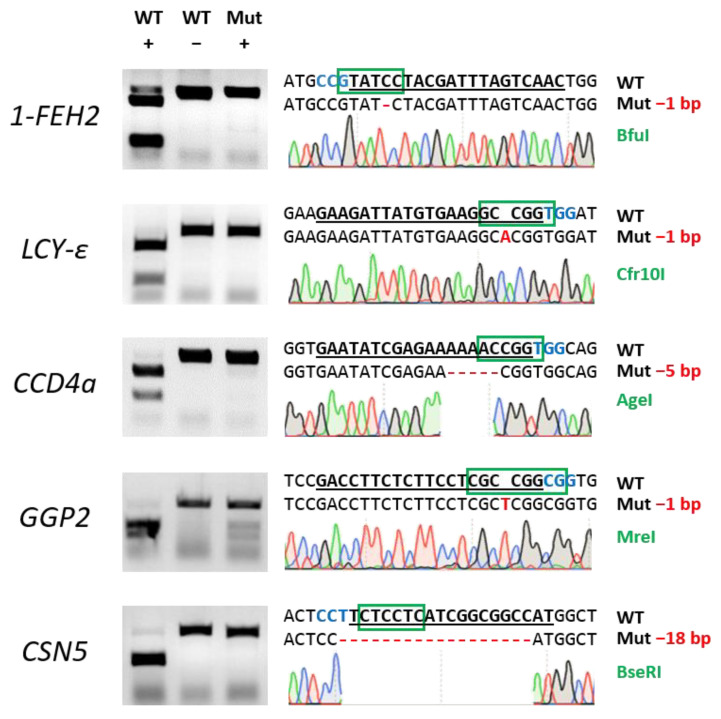
Examples of mutations generated in five target genes: *1-FEH2*, *LCY-ε*, *CCD4a*, *GGP2*, and *CSN5* in lettuce cv. ‘Noga’. (**Left panels**): Targeted gene regions of wild-type (WT) and mutant (Mut) plants were PCR-amplified and digested with the designated restriction enzyme for each individual gene. Digested and non-digested PCR products of WT (WT + and WT −) are shown alongside digested DNA from putative mutants (Mut +). (**Right panels**): Sanger sequencing results of the gRNA target regions for each gene. The gRNA spacer sequence is underlined; the protospacer adjacent motif (PAM) sequence is shown in blue; restriction enzyme names used for each gene are indicated to the right in green font, with their recognition sequences highlighted in green boxes; and indels are shown in red. The size of the indels is indicated next to each sequence. The following restriction enzymes were used for the digestion of the target genes: *1-FEH2*, BfuI; *LCY-ε*, Cfr10I; *CCD4a*, AgeI; *GGP2*, MreI; and *CSN5*, BseRI.

DNA from regenerated plantlets was PCR-amplified using specific primers for each target gene. The product was digested with the corresponding restriction enzyme, and electrophoresis was performed alongside digested (WT +) and undigested (WT −) PCR products from wild-type plants as controls (Figure 3). Gene editing efficiency, expressed as the proportion of undigested edited samples out of all analysed samples, ranged from 23% to 79%, with an average of 47% across the five target genes (Table 1). When calculated relative to the initial number of explants, efficiency ranged from 19% to 75%, with an average of 36%. To confirm the editing events, DNA from putative edited plantlets was sequenced; approximately 30% of the events were bi-allelic or homozygous (Figure 3). PCR analysis of the DsRed gene in offspring seedlings of different gene-edited plants showed no amplification, confirming that TRV expression was transient and that the virus did not pass on to the progeny (Figure S3).

The TRV-based method was also effective for deleting target sequences of approximately 300 bp by cloning two gRNAs into the same pTRV2 vector (Figure S4). Furthermore, it was successfully used to introduce mutations in Noga plants expressing Cas12a, highlighting the versatility of the TRV-based gRNA delivery system (Figure S5).

### 2.2. Increased Fructan Content and Mean Degree of Polymerisation in 1-FEH2 Knockout Lettuce Lines

**Figure 4:**
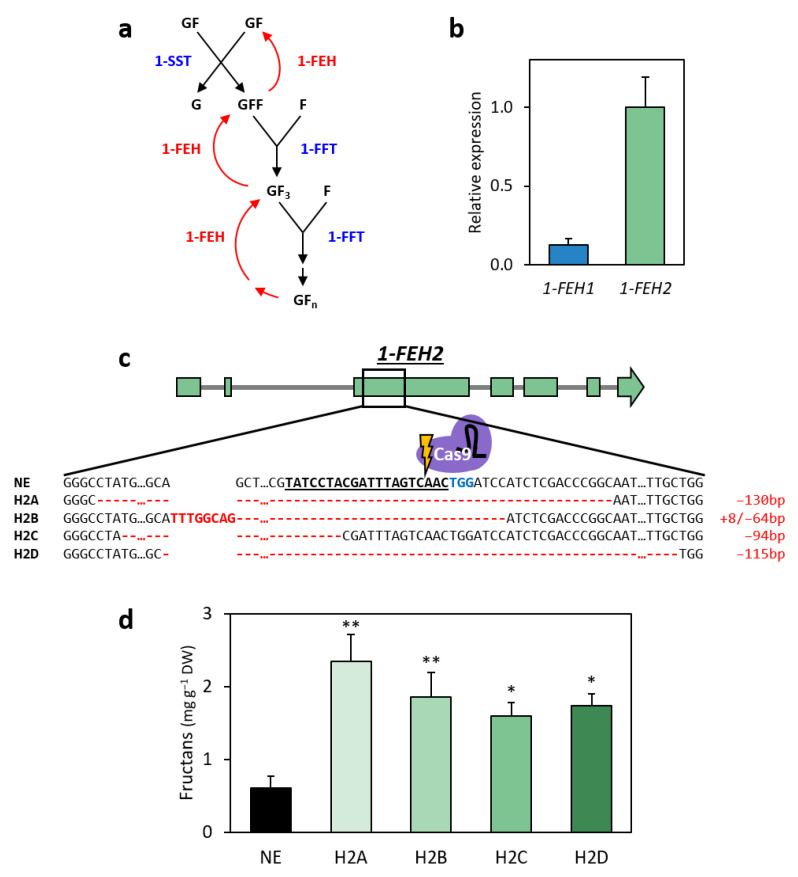
Mutation sequences and fructan content in four independently generated homozygous *1-FEH2* gene-edited lettuce cv. ‘Noga’ lines. (**a**) Schematic representation of fructan biosynthesis in Asteraceae. G = glucose; F = fructose; GF_n_ = fructan with *n* units of fructose; 1-SST = sucrose:sucrose 1-fructosyl transferase; 1-FFT = fructan:fructan 1-fructosyl transferase; and 1-FEH = fructan 1-exohydrolase. (**b**) Relative expression of *1-FEH1* and *1-FEH2* in two-month-old wild-type ‘Noga’ lettuce plants based on RT-qPCR analysis. Gene expression was normalised to the highest expressing gene (*1-FEH2*). Data represent means ± SEM (*n* = 4). (**c**) *1-FEH2* gene structure and sequence in non-edited (NE) plants and mutant lines (H2A, H2B, H2C, and H2D). The gRNA spacer sequence is underlined; PAM sequence is shown in blue. Base indels are indicated in red; the sizes of the indels for each mutant line are displayed to the right. (**d**) Fructan content in two-month-old leaves of *1-FEH2* homozygous mutant lines H2A, H2B, H2C, and H2D compared with non-edited (NE) control plants. Data represent means ± SEM (*n* = 3). Asterisks indicate statistically significant differences compared with NE controls (Dunnett’s test, * *p* ≤ 0.05; ** *p* ≤ 0.01). DW, dry weight.

1-FEH enzymes play a crucial role in the biosynthesis of inulin-type fructans and have been extensively studied in chicory for their impact on inulin accumulation and mean degree of polymerisation (mDP; Figure 4a) [6,25,35,39,45,46]. In lettuce, a close relative of chicory within the Cichorieae tribe of the Asteraceae family, two *1-FEH* homologs were identified (GenBank IDs: XP_023733615.1; XP_023733629.1) following a reciprocal BLAST analysis using chicory 1-FEH protein sequences as queries against the lettuce RefSeq genome [39,45] (Figures S6 and S7). Lettuce 1-FEH1 shares 88.2% amino acid identity with chicory Ci1-FEH1 (Figure S6). A single 1-FEH2 homolog was identified in lettuce, showing 90.2% and 89.3% identity with Ci1-FEH2a and Ci1-FEH2b, respectively (Figure S7). Both lettuce genes are located in adjacent loci on chromosome 5. Consistent with their chicory counterparts, lettuce 1-FEH1 and 1-FEH2 substantially differ in their amino acid sequence (52.4% identity) and exon–intron structure: *1-FEH1* has a large first intron and a very short second intron, characteristic of cell wall invertases, whereas *1-FEH2* has a short first intron and a larger second intron, typical of vacuolar invertases [35]. To determine the expression profiles of these genes in lettuce leaves, RT-qPCR analysis was performed on wild-type ‘Noga’ lettuce. The results show that *1-FEH2* transcript levels were approximately tenfold higher than those of *1-FEH1* (Figure 4b). Similarly, in chicory leaves, mainly *Ci1-FEH2* (and not *Ci1-FEH1*) is expressed [39].

Given that increased inulin content and higher mDP in chicory were associated with sequence variations in *Ci1-FEH2b* but not in *Ci1-FEH1* [35,46], and that transgenic lettuce overexpressing *asnA* with repressed *1-FEH2* activity exhibited increased fructan levels [37], *1-FEH2*, which was also more highly expressed in cv. ‘Noga’ leaves, was selected as the primary knockout target to enhance fructan accumulation. Therefore, a population of TRV-edited *1-FEH2* plants was generated by self-pollination over two generations to confirm mutation inheritance and to produce homozygous mutant lines. These plants grew normally and showed no visible differences compared to non-edited (NE) control lettuce plants under regular growth conditions (Figure S8). Four homozygous gene-edited lines were selected for analysis: H2A (−130 bp deletion), H2B (+8/−64 bp indel), H2C (−94 bp deletion), and H2D (−115 bp deletion; Figure 4c). All deletions resulted in frameshift mutations starting at amino acid 81 or earlier, leading to the introduction of premature stop codons. Fructan analysis in the leaves of these *1-FEH2* mutant lines revealed significant increases, ranging from 2.7- to 3.9-fold compared to NE control plants (Figure 4d). Notably, H2A heterozygous plants with only one mutated *1-FEH2* allele (genotype +−) displayed fructan levels comparable to controls, suggesting that complete inactivation of 1-FEH2 is essential for enhanced fructan accumulation (Figure S9).

**Figure 5:**
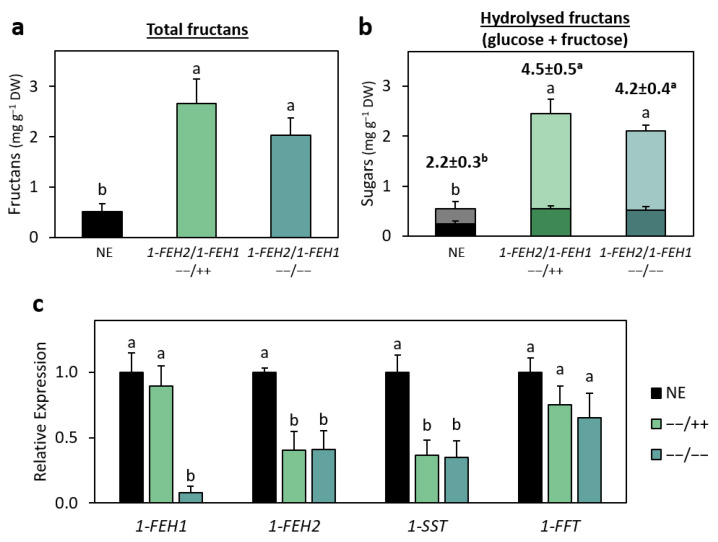
Total fructans, hydrolysed sugars and fructan biosynthesis gene expression in two-month-old *1-FEH2* (−−/++) and *1-FEH2/1-FEH1* (−−/−−) mutant lettuce plants from the H1AxH2A population. (**a**) Total fructan content in leaves of homozygous *1-FEH2* (−−/++) mutants and *1-FEH2/1-FEH1* (−−/−−) double mutants compared with non-edited (NE) control plants. Data represent means ± SEM (*n* = 3). Letters indicate statistically significant differences (one-way ANOVA followed by Tukey’s HSD test, *p* ≤ 0.05). DW, dry weight. (**b**) Glucose (lower part of bar) and fructose (upper part of bar) content, along with mean degree of polymerisation (mDP; indicated above the bars) of hydrolysed fructans extracted from *1-FEH2* (−−/++) mutants, *1-FEH1/1-FEH2* (−−/−−) mutants, and NE control plants. Data represent means ± SEM (*n* = 3). Letters indicate statistically significant differences (one-way ANOVA followed by Tukey’s HSD test, *p* ≤ 0.05). DW, dry weight. (**c**) Relative expression of genes encoding enzymes involved in the fructan biosynthesis pathway in leaves of *1-FEH2* (−−/++) mutants, *1-FEH2/1-FEH1* (−−/−−) mutants, and NE control plants. Data represent means ± SEM (*n* = 4). Letters indicate statistically significant differences (one-way ANOVA followed by Tukey’s HSD test, *p* ≤ 0.05). *1-FEH1* and *1-FEH2* = *fructan 1-exohydrolase 1* and *2*; *1-SST* = *sucrose:sucrose 1-fructosyl transferase*; and *1-FFT = fructan:fructan 1-fructosyl transferase*.

To assess the involvement of *1-FEH1* in fructan production in lettuce, several independent *1-FEH1* knockout mutant lines were generated (Figure S10). The fructan content of these *1-FEH1* knockout lines did not differ significantly from non-edited (NE) control plants, indicating that repression of *1-FEH1* alone does not affect fructan accumulation (Figure S10). To evaluate the effect of *1-FEH1* knockout in the background of a mutated *1-FEH2*, line H1A was crossed with the highest-fructan-content *1-FEH2* knockout line H2A to stack the mutations, and the resulting offspring (H1AxH2A) were self-pollinated. Fructan content analysis of the progeny revealed that leaves from plants with only the *1-FEH2* knockout (−−/++) or a double knockout of *1-FEH1* and *1-FEH2* (−−/−−) exhibited significantly higher fructan levels, showing 5.2-fold and 4.0-fold increases, respectively, compared to NE controls (Figure 5a). However, there was no significant difference between these two genotypes, suggesting that knocking out *1-FEH1* does not further enhance fructan accumulation beyond the effect of *1-FEH2* knockout alone. To assess whether *1-FEH1* influences the degree of polymerisation (DP) of these reserve carbohydrates, extracted fructans were hydrolysed and analysed for their glucose-to-fructose ratio to calculate the mean DP (mDP; Figure 5b). The glucose content in hydrolysed fructans from both mutant genotypes (−−/++ and −−/−−) more than doubled compared to control plants (0.55 and 0.52 mg g^−1^ DW vs. 0.25 mg g^−1^ DW, respectively), while fructose content increased significantly by 6.3-fold and 5.3-fold, respectively. Overall, the mDP of the mutant genotypes increased by 2.3 and 2.0 units, with no significant difference between them. These findings suggest that suppression of *1-FEH2* activity promotes the accumulation of more and longer-chain fructans, whereas *1-FEH1* does not significantly affect either parameter in mutant plants.

To explore how the mutations affect genes related to fructan biosynthesis at the transcriptional level, RT-qPCR was used to analyse the expression of *1-FEH1*, *1-FEH2*, and the upstream biosynthetic genes *1-SST* and *1-FFT* (Figure 5c). *1-FEH2* expression was significantly reduced in both *1-FEH2* mutant genotypes (++/−− and −−/−−) compared to NE control plants. *1-FEH1* transcript levels were significantly reduced in the double mutant (−−/−−) but remained unchanged in the *1-FEH2* (++/−−) single mutant. Additionally, *1-SST* transcript levels were significantly lower in both mutant genotypes (++/−− and −−/−−), hinting at a possible feedback regulation between *1-FEH2* and *1-SST*. The expression of *1-FFT* remained unchanged between NE controls and both mutant genotypes. These findings suggest that the increased fructan content observed in *1-FEH2* mutant genotypes is likely due to reduced 1-FEH2 activity, despite a potential decrease in upstream substrate availability caused by the lower expression of *1-SST*.

## 3. Discussion

Gene editing is a disruptive technology for enhancing crop nutrient profiles, addressing challenges posed by climate change and a growing global population [47]. Lettuce is celebrated globally for its pleasing taste and texture, making it one of the most widely consumed leafy vegetable worldwide [18]. However, it is generally less nutritionally rich than other leafy greens, particularly in fibre content [19,20,21,22]. As a member of the Asteraceae family, lettuce produces inulin-type fructans, prebiotic fibres associated with improved gut health, immune system support and reduced risk of chronic diseases, albeit in relatively small amounts [26,27,33,34]. Consuming dietary fibres, such as fructans, in their natural vegetal context is suggested to confer additional health benefits compared to processed dietary supplements [6]. Establishing an efficient and versatile gene editing system in lettuce to enhance nutrients, including fructan content, aligns with contemporary consumer demand for functional foods and offers an avenue to address the global “fibre gap” [5,48].

A major limitation of gene editing efficiency is variability in CRISPR component expression across transformation events, which poses particular challenges when targeting non-model and/or recalcitrant plants [49,50]. Despite tissue culture-related limitations, random transgene integration, possible gene silencing, and unpredictable expression patterns, *Agrobacterium*-mediated transformation in tissue culture approaches are the most commonly practiced methods for genome editing in laboratories globally [2].

In this study, we utilised the transient expression of gRNA via TRV in lettuce plants stably expressing Cas9. This approach achieved high editing efficiencies across several genes (up to 79%), significantly surpassing rates reported for *Agrobacterium*-mediated gene editing in lettuce [51,52,53]. The method eliminates the need for antibiotic selection during the gene editing stage, enhancing cell viability, simplifying tissue culture processes, and reducing regeneration times [54,55,56]. As TRV is an RNA virus, the progeny of the edited plants does not contain the viral genes, addressing potential regulatory concerns [38].

The separation of Cas9 integration from TRV-mediated gRNA delivery ensures consistent Cas9 activity and enables reinoculation of Cas9-expressing plants with different gRNAs to target additional genes, facilitating efficient stacking of traits over multiple cycles without requiring selection via antibiotics. By minimising variability associated with differences in Cas9 integration sites, this method also supports reliable comparisons of various gRNA activities, a factor greatly affecting gene editing efficiency [44]. The versatility of the system was demonstrated here by two additional capabilities: its ability to delete targeted genomic fragments using dual gRNAs and to introduce edits via Cas12a-compatible gRNAs. Future adaptations could broaden its application to advanced genome editing techniques, such as base and prime editing [57]. Once the desired gene editing outcome is achieved using this system, the constitutively expressed *Cas9* can be removed through conventional breeding, resulting in a transgene-free, genome-edited product. This approach allows for the commercialisation of enhanced genome-edited lettuce as non-GMO in countries where regulations focus on the final product rather than the method used to obtain it. Such regulations are currently in place in North and South America, Australia, Japan, and the Philippines, with a growing global trend toward this approach [58,59].

The practical utility of the TRV-based gene editing system for biofortification of lettuce was demonstrated in suppressing 1-FEH2 activity, a key enzyme in fructan metabolism responsible for its degradation. In all four *1-FEH2* mutant lines, the frameshift mutations occur upstream of two key catalytic residues (D185 and E239) required for enzymatic function in the closely related chicory 1-FEH2b [35]. Knocking out *1-FEH2* resulted in a significant increase in fructan content, with levels rising up to 5.2-fold compared to non-edited control plants, accompanied by an increase in fructan mDP. These results align with the findings in chicory, where loss-of-function mutations in *Ci1-FEH2b* similarly led to increased inulin content and mDP [35]. Knocking out *1-FEH1* in lettuce had no significant effect on fructan content, indicating that *1-FEH1* is not a major contributor to fructan degradation under the growth conditions applied in this study.

Fructans serve a dual role in plants, functioning as storage carbohydrates and contributing to environmental stress tolerance [60,61,62,63]. Interestingly, in chicory, *1-FEH* genes are induced in response to environmental cues such as cold exposure and defoliation [39,45,64]. In lettuce, moderate water deficit induced an approximately 2.5-fold fructan, enhancing tolerance to osmotic imbalances and maintaining structural integrity under draught and cold stress [28]. Similarly, a transgenic expression of lettuce *1-SST* in tobacco led to the production of short-chain fructans, improving cold stress tolerance [65]. These findings suggest that the increased fructan content in the gene-edited lettuce may enhance its resilience to water stress and cold exposure, potentially extending shelf life by retaining its crisp texture in post-harvest cold storage. Testing gene-edited lettuce lines under various growth settings could reveal additional roles for *1-FEH1* and *1-FEH2* in carbohydrate metabolism and cellular homeostasis, with potential applications for enhancing pre-harvest quality and post-harvest crispness.

Short-chain fructans, such as those naturally found in lettuce, are rapidly fermented by gut microbiota in the proximal colon, producing beneficial short-chain fatty acids. In contrast, longer-chain fructans undergo slower fermentation, allowing them to reach the distal colon, where they continue to support prebiotic activity through sustained metabolism [66,67]. In this study, the mDP of *1-FEH2* gene-edited lettuce increased by 2.3 units compared to non-edited control plants. This increase suggests a shift toward more gradually fermentable fructans, potentially enhancing gut microbiota diversity and extending prebiotic effects further along the colon. According to Van Laere and Van den Ende, an absence of 1-SST activity causes 1-FFT to transfer fructosyl units to sucrose or free fructose, reducing mDP in the context of chicory inulin [23]. Preventing the autumnal decline of 1-SST activity in chicory through transgenic over-expression led to a 20% increase in mDP [68]. Analysis of lettuce *1-FEH2* mutant transcript levels revealed that *1-SST* levels were decreased, which may have limited the flux towards fructan biosynthesis, suggesting a similar regulatory mechanism in lettuce that potentially balances fructan synthesis and degradation. Future studies combining *1-FEH2* knockout with *1-SST* upregulation may allow further enhancement of fructan content and mDP in lettuce, potentially amplifying its prebiotic benefits.

## 4. Materials and Methods

### 4.1. Plant Material and Growth Conditions

Romaine lettuce (*Lactuca sativa* L. cv. ‘Noga’) seeds were kindly provided by Hazera Ltd. Seeds were surface-sterilised by immersing them in 70% ethanol for 60 s, followed by treatment with 2.5% sodium hypochlorite for 15 min while gently spinning. The sterilised seeds were then sown on germination medium containing 0.25× Murashige and Skoog (MS) basal salts, 0.75% AGA03 agar (Formedium, Norfolk, UK), and 1% sucrose. Seedlings with two to three leaves were sampled for DNA extraction (if required) and transferred to cell trays filled with gardening soil mix for an acclimatisation period of approximately two weeks. The plants were subsequently transplanted into 12 cm cylindrical pots and cultivated in a greenhouse under controlled conditions of 26 °C/20 °C day/night temperatures and a 16 h light/8 h dark photoperiod. Incandescent bulbs (120 V, 16 lm/W) provided additional lighting as required.

To ensure homozygosity and eliminate the possibility of chimerism, all mutant lines were selfed for three generations before analysis. Individual progeny plants were subjected to Sanger sequencing, and sequencing chromatograms were carefully examined.

Cross-pollination was conducted by sterilising unopened female-designated flower buds just after dawn with 5–10 µL of double-distilled water, applied using a pipette tip. Once the buds opened and dried completely, pollen from male-designated flowers was gently applied to the sterilised female flowers to ensure fertilisation. Fertilised flowers were marked for seed collection.

To generate the double-mutant H1AxH2A population, the *1-FEH1* knockout line H1A was crossed with the *1-FEH2* knockout line H2A. F₁ progeny was self-pollinated, and F₂ plants were screened via sequencing to identify homozygous *1-FEH2* mutants or double mutants (*1-FEH1* and *1-FEH2*).

### 4.2. Plasmid Construction and gRNA Design

Nuclease expression constructs (pCGN-UbqP:hCas9 and pCGN-UbqP:Cas12a) were generated as follows: the human codon-optimised Cas9 gene (*hCas9* [69], kindly provided by Dr. Moshe Fleischman) or LbCas12a gene ([70]; synthesised by Twist Bioscience, San Francisco, CA, USA) was placed under the control of the *Arabidopsis thaliana* ubiquitin promoter 10 [71]. These were combined with the *nptII* gene driven by the CaMV 35S promoter and cloned into the binary vector pCGN [72]. The mGUS-gRNA expression construct (pRCS-mGUS/gRNA) was generated as described previously [15,38].

Target gene sequences (*1-FEH1*, *1-FEH2*, *LCY-ε*, *CCD4a*, *GGP2*, *CSN5*, and *HMG-1* [GenBank ID: XM_023872940.1]) were PCR-amplified from DNA extracted from cv. ‘Noga’ plants based on the published lettuce genome [73], and the sequences were verified by Sanger sequencing. Guide RNAs (gRNAs) compatible with Cas9 or Cas12a nucleases were designed using CRISPOR, prioritising high specificity to minimise off-target editing [44]. The GenBank accession numbers of the pTRV1 and pTRV2 [74] used in this study are AF406990 and AF406991, respectively. Each gRNA was cloned into the pTRV2 vector under the control of TRV’s coat protein subgenomic promoter, along with the *DsRed* fluorescence marker gene driven by the pea early browning virus subgenomic promoter, to create gRNA expression constructs (pTRV2-gRNA-DsRed). These constructs were then used to transform lettuce explants stably expressing either Cas9 or Cas12a. For agroinoculation, pTRV1 and recombinant pTRV2 vectors were mobilised into Agrobacterium strain AGL0 as previously described [13,75].

### 4.3. Tissue Culture and Transformation

*A. tumefaciens* strain AGL0 [75] was used for all transformations. Bacterial cultures were incubated overnight in the dark at 28 °C and grown in LB Broth Lennox medium (Formedium, Norfolk, UK) supplemented with 100 μM acetosyringone (Sigma, St. Louis, MO, USA) and the appropriate antibiotic: pCGN, 40 μg/mL gentamycin; pRCS2, 300 μg/mL spectinomycin and 200 μg/mL streptomycin; and pTRV1 and pTRV2, 50 μg/mL kanamycin. Bacterial cells were centrifuged at 6000× *g* for 5 min at room temperature, washed, and resuspended with 10 mM MgCl₂ containing 100 μM acetosyringone to an OD_600_ of 0.5. Cotyledons from six-day-old seedlings were dissected to include the petiole and immersed in *A. tumefaciens* solution for 10 min at room temperature. Inoculated cotyledons were placed between two filter papers on a co-cultivation medium (1× MS, 3% sucrose, 100 μM acetosyringone) and incubated in the dark for two days. Cotyledons were then transferred to regeneration medium (1× MS, 3% sucrose, 300 mg/L carbenicillin). For regeneration optimisation assays, varying concentrations of 6-benzylaminopurine (BA) and 1-naphthaleneacetic acid (NAA) were tested (Table S1). In subsequent transformations, Regeneration Medium 6 (0.5 mg/L BA, 0.04 mg/L NAA) was used. For regenerating Cas9- or Cas12a-expressing plants, 50 mg/L kanamycin was included as a selection agent. No antibiotic selection was used in gRNA transformation procedures. Cotyledons were subcultured onto fresh medium every 5–7 days until distinct plantlets developed. Regenerated plantlets were separated from the calli and transferred to rooting medium (1× MS, 3% sucrose).

### 4.4. Leaf Infiltration and Histochemical GUS Staining

Leaves of 14-week-old regenerated cv. ‘Noga’ plants were scratched on the abaxial side using a needle and then infiltrated with a solution of *A. tumefaciens* harbouring the mGUS-gRNA expression construct pRCS-mGUS/gRNA. Three leaves were inoculated per plant. The leaves remained attached to the plants and were examined for β-glucuronidase (GUS) activity five days post-inoculation.

Histochemical GUS staining was performed following a previously published protocol [76]. An X-Gluc solution was prepared by dissolving 5-bromo-4-chloro-3-indolyl β-D-glucuronic acid powder in dimethylformamide (DMF) to a concentration of 3.33% (*w*/*v*). An iron-cyanide solution was prepared by dissolving 21.1 mg/L potassium ferricyanide and 16.5 mg/L potassium ferrocyanide in double-distilled water (DDW). To prepare the staining buffer, 100 mM sodium phosphate (pH 7.5), 10 mM EDTA, and 0.1% Triton X-100 were combined. The X-Gluc and iron-cyanide solutions were then added to the buffer to final concentrations of 0.0025% and 0.001% (*v*/*v*), respectively. Infiltrated leaf samples were submerged in the staining solution for 16 h at 37 °C. Subsequently, the samples were transferred to 70% ethanol until all chlorophyll was extracted, allowing the blue spots produced by GUS activity to become visible.

### 4.5. Generation of Gene-Edited Plants

Cotyledon explants were transformed with pTRV1 and pTRV2-gRNA-DsRed constructs as described above. To confirm successful TRV infiltration, transformed cotyledons were examined under a fluorescence binocular microscope (Nikon SMZ18; Nikon, Tokyo, Japan; https://www.nikon.com) 4–6 days post-inoculation. Infiltration was considered successful when DsRed fluorescence covered more than 90% of the leaf surface. Viable regenerated plantlets, at least 10 mm in length, were sampled for DNA extraction. Target regions were amplified by PCR using gene-specific primers, and 60 ng of the PCR product was digested overnight with restriction enzymes following the manufacturer’s instructions. Digested samples were analysed by gel electrophoresis alongside digested and undigested wild-type (WT) controls. Putative gene-edited samples, identified by bands at similar positions to undigested WT controls, were excised from the gel, cloned into the pJET vector (CloneJET PCR Cloning Kit, Thermo Fisher Scientific, Waltham, MA, USA), and confirmed by Sanger sequencing. The following restriction enzymes were used for the digestion of the target genes: *1-FEH1*, BseDI or MbiI; *1-FEH2*, BciVI; *LCY-ε*, Cfr10I; *CCD4a*, AgeI; *GGP2*, MreI; and *CSN5*, BseRI or MbiI (New England Biolabs, Ipswich, MA, USA; Thermo Fisher Scientific, Waltham, MA, USA).

### 4.6. Fructan and Sugar Extraction and Quantificaiton

Two to three fully expanded leaves, with a combined weight of at least 20 g, were harvested from two-month-old plants. The leaves were freeze-dried via lyophilisation until completely dehydrated, then ground into a fine powder using a mortar and pestle. Fructan and sugar quantification were carried out following the protocols outlined in the K-FRUC and K-FRUGL enzymatic assay kits (Megazyme, Wicklow, Ireland; AOAC methods 999.03 and 985.09, respectively). In brief, fructans were extracted from 200 mg of lyophilised leaf powder in 5 mL of double-distilled water by boiling for 15 min, followed by cooling and centrifugation to remove insoluble material. To eliminate interference from sucrose and starch, enzymatic hydrolysis was performed to convert reducing sugars into sugar alcohols. Fructans in the extract were hydrolysed into glucose and fructose using recombinant inulinase and levanase enzymes. The resulting monosaccharides were quantified with a *p*-hydroxybenzoic acid hydrazide (PAHBAH)-based colorimetric assay, and absorbance was measured at 410 nm using a Multiskan SkyHigh spectrophotometer (ThermoFisher Scientific). To quantify glucose and fructose content, the same lysate was subjected to a hexokinase-based enzymatic assay. In this process, glucose and fructose were phosphorylated, and the production of NADPH was monitored at 340 nm. Calibration curves derived from glucose and fructose standards were used to calculate their respective concentrations.

The mean degree of polymerisation (mDP) of the fructans was calculated using the measured fructose and glucose concentrations according to the following formula:

$$mDP=\frac{Fructose}{Glucose}+1$$

### 4.7. RNA Extraction and Quantitative Real-Time PCR (RT-qPCR)

Total RNA was extracted from approx. 200 mg of leaf tissue collected from two-month-old plants. The tissue was ground in liquid nitrogen, and RNA was isolated using the Tri-Reagent kit (Sigma, St. Louis, MO, USA), followed by treatment with RNase-free DNase I (ThermoFisher Scientific) to eliminate genomic DNA contamination. First-strand cDNA was synthesised from total RNA using an oligo(dT) primer and ImProm-II reverse transcriptase (Promega, Madison, WI, USA) according to the manufacturer’s instructions.

Quantitative real-time PCR (qRT-PCR) was performed for 35 cycles (initial denaturation at 95 °C for 5 min, followed by 35 cycles of 95 °C for 5 s and 60 °C for 35 s) using 2X qPCRBIO SyGreen Blue Mix Hi-ROX (PCR Biosystems, London, UK). Reactions were conducted on a Rotor-Gene Q cycler (Qiagen, Hilden, Germany) and a CFX Opus 384 Real-Time PCR System (Bio-Rad, Hercules, CA, USA). Expression levels were normalised using *ACTIN2* (*ACT2*) and *TAP42*-interacting protein of *41 kDa* (*TIP41*) as reference genes [77]. Relative quantification of target gene expression was calculated using the 2^−ΔΔCT^ method as described [78]. Primer sequences are provided in Table S2.

### 4.8. Sequence Alignments and Statistics

Homologous sequences were identified using BLAST+ 2.11.0. Sequence alignments were performed using the Clustal Omega 1.2.4 multiple sequence alignment by EMBL-EBI, using default parameters [79]. Statistical analyses were conducted using JMP Pro 17 software (SAS Institute, Cary, NC, USA).

## 5. Conclusions

This study demonstrates the successful use of a TRV-based gRNA delivery system in lettuce, overcoming key challenges of conventional CRISPR/Cas9 methods, including inefficient reagent delivery and labour-intensive tissue culture processes. The approach was validated through the biofortification of a commercial lettuce cultivar, significantly enhancing fructan quantity and quality. These advancements position lettuce as a functional food that aligns with consumer demand for health-focused products and dietary guidelines promoting fibre consumption. The efficient gene editing approach applied here to target fructan content in lettuce could be applied to stack additional traits in this vegetable as well as be applied to other crops.

## Figures and Tables

**Table 1:** Regeneration and gene editing efficiencies for five target genes in lettuce cv. ‘Noga’ using the TRV-mediated editing system.

Target Gene	Regeneration Efficiency ^1^	Editing Efficiency (%/Regenerated) ^2^	Editing Efficiency (%/Initial Explants) ^3^
*1-FEH2*	81.0%	32.4%	26.2%
*LCY-ε*	48.8%	38.5%	18.8%
*CCD4a*	58.0%	35.0%	20.3%
*GGP2*	64.2%	58.8%	37.7%
*CSN5*	95.0%	78.9%	75.0%
**Average**	**69.4%**	**48.7%**	**35.6%**

^1^ Calculated as the percentage of plantlets regenerated from the initial number of explants. ^2^ Calculated as the percentage of gene-edited plantlets from the number of regenerated plantlets. ^3^ Calculated as the percentage of gene-edited plantlets from the number of the initial number of explants.

## Data Availability

The original contributions presented in this study are included in the article/Supplementary Materials. Further inquiries can be directed to the corresponding author(s).

## Supplementary Materials

The following supporting information can be downloaded at: https://www.mdpi.com/article/10.3390/ijms26062594/s1.
