# Supplementary material for: Viral-Based Gene Editing System for Nutritional Improvement of Fructan Content in Lettuce
Source: Int J Mol Sci. 2025 Mar 13;26(6):2594. doi: 10.3390/ijms26062594 (PMC11942539; doi:10.3390/ijms26062594)
Supplement: Supplementary file 1 [file ijms-26-02594-s001.zip › ijms-3437765-supplementary.pdf]

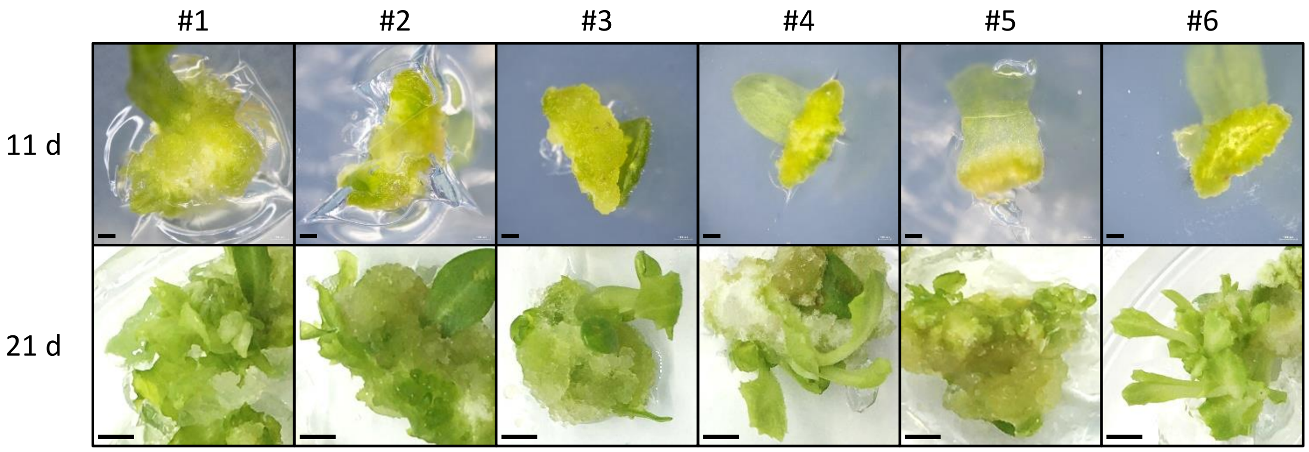

Supplementary Figure S1: Regeneration of lettuce cv. 'Noga' cotyledons cultured on six regeneration media with varying cytokinin (6-benzylaminopurine, BA) and auxin (1-naphthaleneacetic acid, NAA) concentrations and ratios. Top row: Cotyledons after 11 days in each regeneration medium, showing the cut surface where callus forms. Bar = 1 mm. **Bottom row**: Cotyledons after 21 days, displaying callus formation and any regenerated plantlets. Bar = 5 mm. Regeneration media are numbered 1–6, corresponding to Table S1.

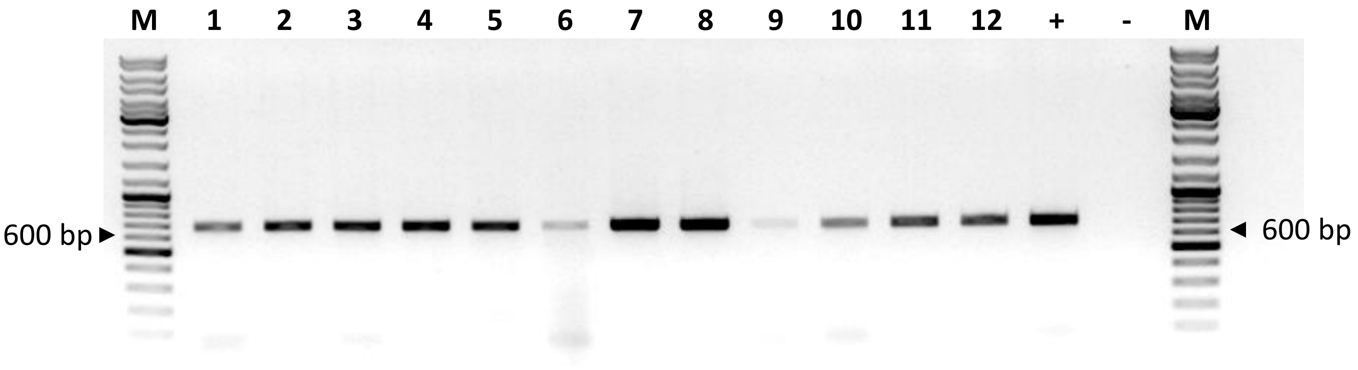

Supplementary Figure S2: PCR confirmation of *Cas9* transformation in putative transgenic lettuce cv. 'Noga' plants. PCR products amplified using primers targeting the *Cas9* gene in putative transgenic plantlets to verify successful transformation. M, molecular marker; +, positive control (pCGN-UbqP:hCas9 construct); -, negative control (wild-type 'Noga' lettuce plant).

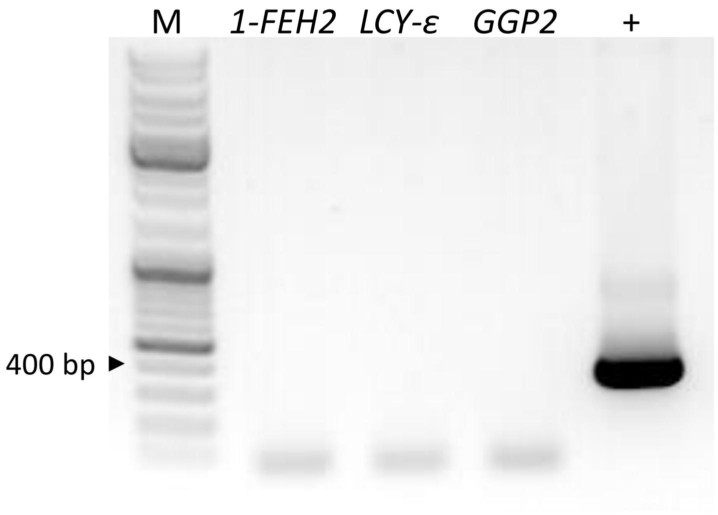

Supplementary Figure S3: PCR analysis of *DsRed* gene in randomly selected progeny of gene-edited plants. Lane 1: Molecular marker (M); Lanes 2-4: PCR products from DNA of three T_2 progeny plants mutated in either *1-FEH2*, *LCY-ε* or *GGP2*, respectively; Lane 5: pTRV2-gRNA-DsRed plasmid (positive control, +).

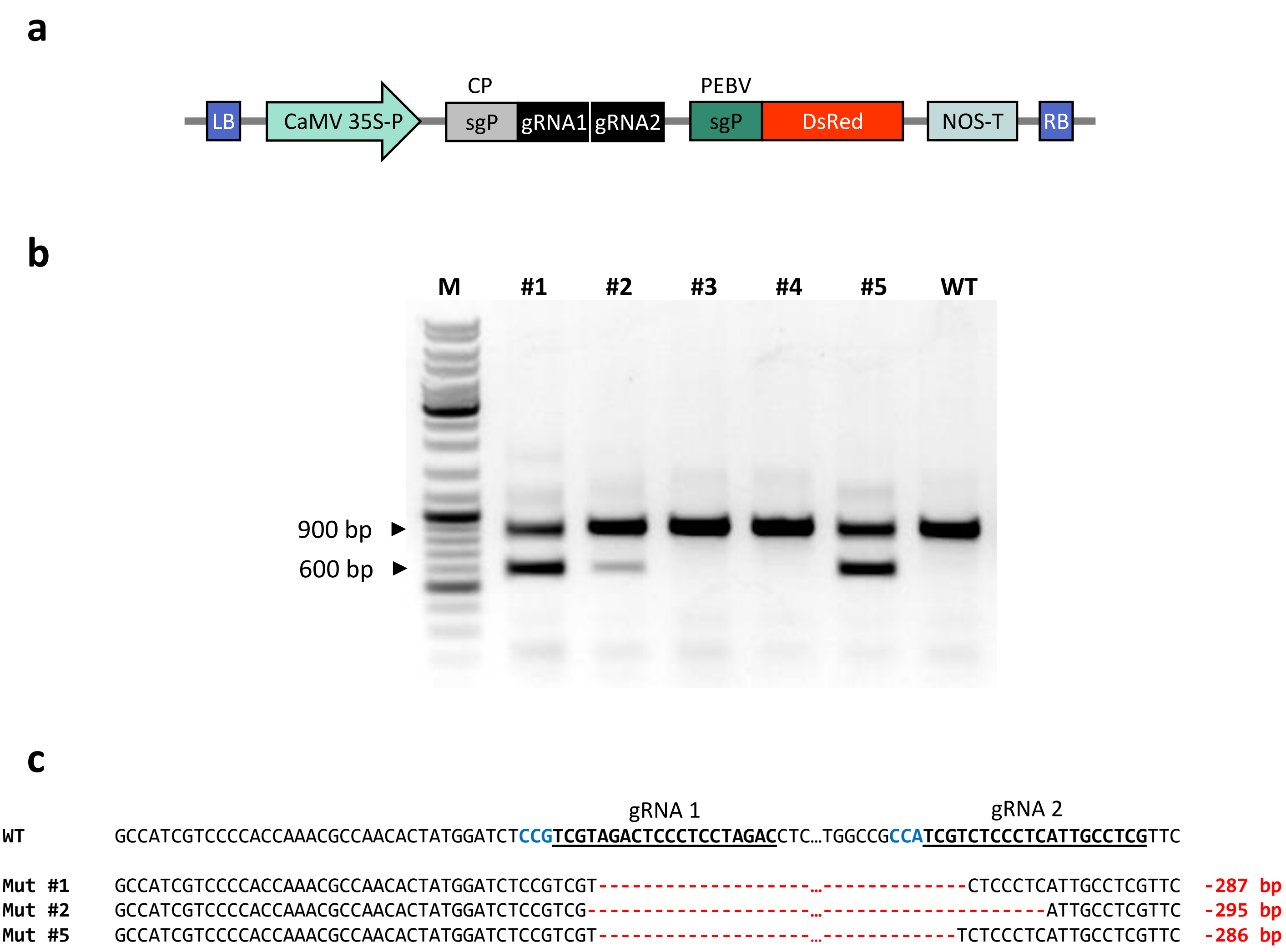

Supplementary Figure S4: Deletion of a ~300 bp fragment from the *HMG-1* gene in lettuce cv. 'Noga' using TRV-mediated gene editing with dual gRNAs.
(a) Schematic representation of the T-DNA region of the vector pTRV2-gRNA1-gRNA2-DsRed, used for TRV–mediated gene editing in Cas9-expressing plants. LB = left border; 35S-P = Cauliflower Mosaic Virus (CaMV) 35S promoter; sgP = sub-genomic promoter of TRV coat protein (CP); gRNA = single-guide RNA (scaffold + spacer); PEBV = pea early browning virus; NOS-T = nopaline synthase terminator; RB = right border. Two gRNA spacer sequences were designed to flank a 304 bp region of interest.
(b) PCR amplification of DNA extracted from regenerated plantlets transformed with pTRV2-gRNA1-gRNA2-DsRed, targeting the *HMG-1* gene. The expected amplicon size for the wild-type (WT) plant is 881 bp. Shorter PCR products indicate large deletions in target sequences.
(c) Sanger sequencing of WT and mutant plants showing the targeted region in the *HMG-1* gene. Both gRNA spacer sequences are underlined; protospacer adjacent motifs (PAM) are shown in blue; deletions are marked in red. The size of each deletion is indicated to the right of the sequence.

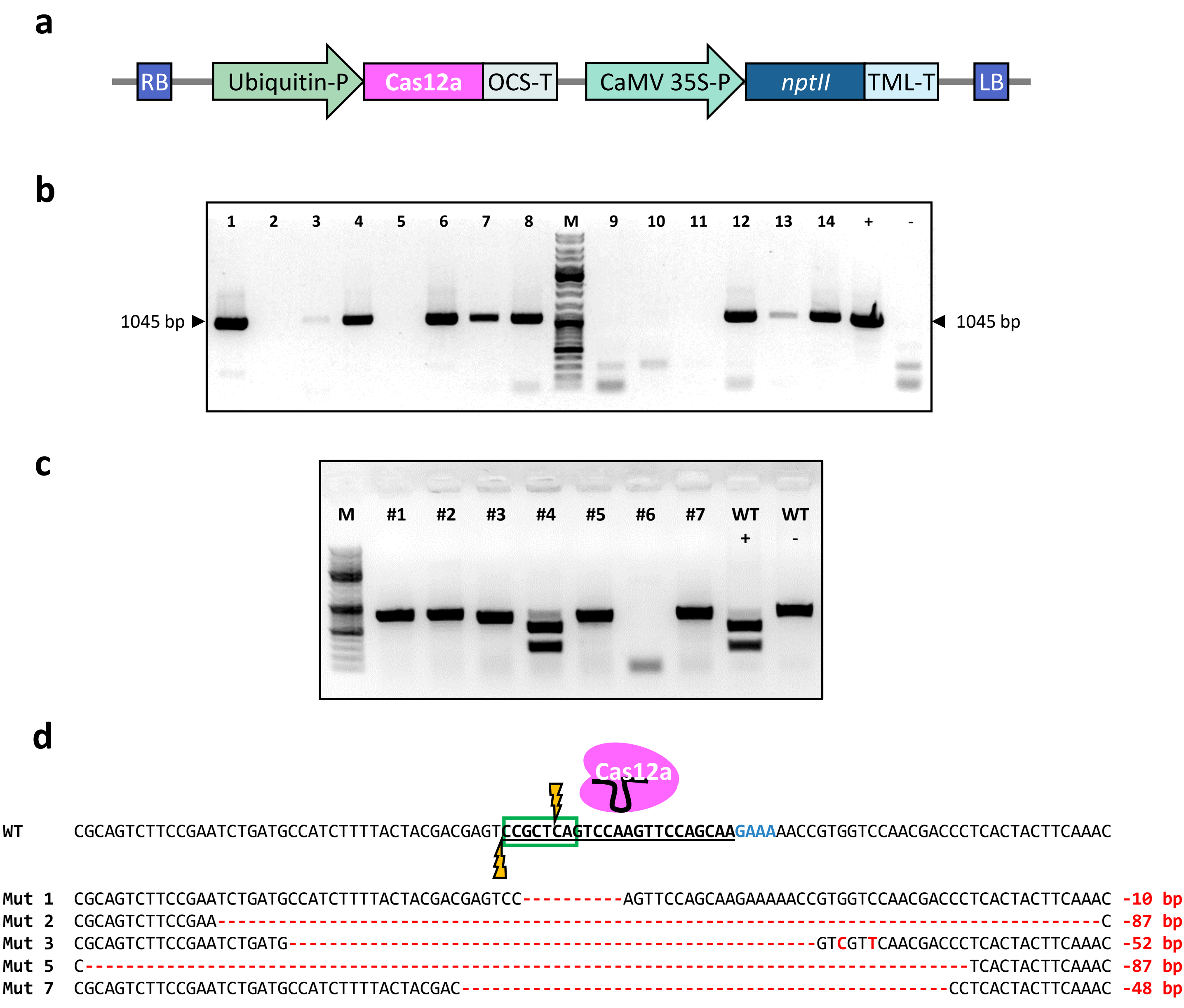

Supplementary Figure S5: Generation of *CSN5* mutant lettuce cv. 'Noga' plants using Cas12a and the TRV system.
(a) Schematic representation of the T-DNA region of the binary plasmid pCGN-UbqP:Cas12a, used to generate Cas12a-expressing lettuce plants. RB = right border; LB = left border; Ubiquitin-P = *Arabidopsis thaliana* Ubiquitin 10 promoter; Cas12a = Cas12a gene; OCS-T = octapine synthase terminator; CaMV 35S-P = Cauliflower Mosaic Virus (CaMV) 35S promoter; *nptII* = neomycin phosphotransferase II gene; TML-T = *Agrobacterium tumefaciens* tumour morphology large (TML) gene terminator.
(b) PCR confirmation of Cas12a gene in putative transgenic lettuce plants. PCR products were amplified using primers targeting the Cas12a gene. M, molecular marker; +, positive control (pCGN-UbqP:Cas12a construct); -, negative` control (wild-type 'Noga' lettuce plant).
(c) PCR-amplified *CSN5* gene region of regenerated plantlets digested with MbiI, alongside digested and non-digested wild-type PCR products as controls (WT + and WT -, respectively).
(d) Sanger sequencing of WT and several mutant plants showing the edited targeted region in the *CSN5* gene. The gRNA spacer sequence is underlined; the protospacer adjacent motif (PAM) is in blue; the MbiI restriction enzyme recognition sequence is enclosed in a green rectangle; deletions are shown in red. The size of each deletion is indicated to the right of the sequence.

```
Ci1-FEH1        MCSERRVKEILGIWVLSLCLVWVQNGVGVHSSSPTEESQPYRTGFHFQPPKNWINDPNGP    60
Ls1-FEH1        MCLKIWVKELLCVWVLSLCLVWVLNGVGVHASSPTDELQPYRSGFHFQPPKNWINDPNGP    60
                ** :  ***:* :********** ******:****:* ****:*****************

Ci1-FEH1        MYFNGVYHLFYQYNPYGPLWGNISWGHSISYDLVNWFLLEPALSPKEPYDINGCLSGSAT    120
Ls1-FEH1        MYFNGVYHLFYQYNPYGPLWGNISWGHSISYDLVNWFLLEPALTPKEPYDINGCLSGSTT    120
                *******************************************:**************:*

Ci1-FEH1        ILPGPRPIILYTGQDVNNSQVQNLAFPKNLSDPLLKEWIKWSGNPLLTPVDDIKAGQFRD    180
Ls1-FEH1        ILPGPQPIILYTGQDLNNSQVQNLAFPKNLSDPLLKDWIKWSGNPFLTPIDDIKPGQFRD    180
                *****:*********:********************:********:***:**** *****

Ci1-FEH1        PSTAWMGPDGKWRIVIGSEIDGHGTALLYRSTNGTKWIRSKKPLHFSSKTGMWECPDFYP    240
Ls1-FEH1        PSTAWIGPDGKWRIVIGSEINGHGTALLYHSRNGTSWTRSKNPLYFSSKTGMWECPDFYP    240
                *****:**************:********:* ***.* ***:**:***************

Ci1-FEH1        VTNGDKKGLDTSVQGNNTLHVLKVSFNSREYYVIGTYDPIKDKFSVVTNDFMVSNTQFQY    300
Ls1-FEH1        VSSGDKKGLDTSFEGNNTIHVLKASFNNREYYVIGNYDPIKDQFFVIGNDFMVSNTQFQY    300
                *:.*********.:****:****.***.*******.******:* *: ************

Ci1-FEH1        DYGRYYASKSFYDSVNQRRVIWGWVNEGDSESDAVKKGWSGLQSFPRSIWLSNNRKQLVQ    360
Ls1-FEH1        DYGRFYASKSFYDGANQRRVLWGWVNEGDSQSDDVKKGWSGLQSFPRSIWLSNNRKQLVQ    360
                ****:********..*****:*********:** **************************

Ci1-FEH1        WPVDEILKLRTKQVNITNRELAAGELLKIPSITASQADVEVSFSLTNLTEIELIDSEVVD    420
Ls1-FEH1        WPVDEIQKLRTKRFNITNRELPGSVLLKIPSITASQADVEVSFSLTNLSEIELIDSEAID    420
                ****** *****:.******* .. ***********************:********.:*

Ci1-FEH1        PQLLCAQKNVSISGKFGPFGMLILASKNLTEQTAVFFRVFKGPNKFLVLMCSDQSRSSIA    480
Ls1-FEH1        PQLLCSQKNASISGRFGPFGLLILASKNLTEQTAVFFRVFKGPNKFLVLMCSDQSRSSIA    480
                *****:***.****:*****:***************************************

Ci1-FEH1        QEVDKSIYGAFLDLDPLHEKIPLRSLIDHSIVESFGGEGIACITSRVYPKLAINEQAELY    540
Ls1-FEH1        QEVDKSIYGAFLDLDPLHEKIRLRSLIDHSIVESFGGKGLSCITARVYPKLAINEQAELY    540
                ********************* ***************:*::***:***************

Ci1-FEH1        VFNNGTQSVTMSTLNAWSMKRAQIVPIG    568
Ls1-FEH1        VFNNGTQSVTISTLNAWSMKEAQIVPIG    568
                **********:*********.*******

#  Percent Identity  Matrix - created by Clustal2.1
     1: Ci1-FEH1    100.00   88.20
     2: Ls1-FEH1     88.20  100.00
```

Supplementary Figure S6: Amino acid sequence alignment and percent identity of 1-FEH1 proteins from chicory (Ci1-FEH1) and lettuce (Ls1-FEH1). Sequence alignment was performed using Clustal Omega (EMBL-EBI). Conserved residues are indicated with asterisks (*); strong and weak similarity are marked with colons (:) and periods (.), respectively.

```
Ls1-FEH2        MNKLLSSFLALCFLIIFQETGHTKVAGRNLKDEILLLNQQIEQPYRTGYHFQPPSNWMND    60
Ci1-FEH2a       MKKSLSSFIVLCFLVIILETGRVKATSRNLNDVIMLANQQIEQPYRTGYHFQPPSNWMND    60
Ci1-FEH2b       MKKSLSSFIALCFLVIVLETGRVKATSRDLNDVILLANQQIQQPYRTGYHFQPPSNWMND    60
                *:* ****:.****:*. ***:.*.:.*:*:* *:* ****:******************

Ls1-FEH2        PNGPMLYKGVYHFFYQYNPYAATFGDIIIWAHAVSYDLVNWIHLDPAIYPTQEADIKSCW    120
Ci1-FEH2a       PNGPMLYQGVYHFFYQYNPYAATFGDVIIWGHAVSYDLVNWIHLDPAIYPTQEADSKSCW    120
Ci1-FEH2b       PNGPMLYQGVYHFFYQYNPYAATFGDVIIWAHAVSYDLVNWIHLDPAIYPTQEADSKSCW    120
                *******:******************:***.************************ ****

Ls1-FEH2        SGSATILPGNIPAMLYTGSDSKSRQVQDLAWPKNLSDPFLREWVKHPKNPLITPPEGVKD    180
Ci1-FEH2a       SGSATILPGNIPAMLYTGSDSKSRQVQDLAWPKNLSDPFLREWVKHPKNPLITPPEGVKD    180
Ci1-FEH2b       SGSATILPGNIPAMLYTGSDSKSRQVQDLAWPKNLSDPFLREWVKHPKNPLIIPPEGVKD    180
                **************************************************** *******

Ls1-FEH2        DCFRDPSTAWLGDDGVWRIVVGGDRDNNGMAFLYQSTDFVNWKRYEQPLSSAVATGTWEC    240
Ci1-FEH2a       DCFRDPSTAWLGPDGVWRIVVGGDRDNNGMAFLYQSTDFVNWKRYDQPLSSADATGTWEC    240
Ci1-FEH2b       DCFRDPSTAWRGPDGVWRIVVGGDRDNNGMSLLYQSTDFVNWKRYDQPLSSAIATGTWEC    240
                ********** * *****************::*************:****** *******

Ls1-FEH2        PDFYPVPLNSTNGLDTSVNTGSFKHVMKAGFEGHDWYTIGTYSSDHENFLPQNGLSLTGS    300
Ci1-FEH2a       PDFYPVPLNSTNGLDTSVYGGSVRHVMKAGFEGHDWYTIGTYSPDRENFLPQNGLSLTGS    300
Ci1-FEH2b       PDFYPVPLNSTNGLDTSVYGGSVRHVMKAGFEGHDWYTIGTYSPDRENFLPQNGLSLTGS    300
                ******************  **.:****************** *:**************

Ls1-FEH2        TLDLRYDYGQFYASKSFFDDAKKRRVLWAWVPETDSQEDDIEKGWAGLQSFPRALWIDRS    360
Ci1-FEH2a       TLDLRYDYGQFYASKSFFDDAKNRRVLWAWVPETDSQADDIEKGWAGLQSFPRALWIDRN    360
Ci1-FEH2b       TLDLRYNYGQFYASKSFFDDAKNRRVLWAWVPETDAPEDDIEKGWAGLQSFPRALWIDRS    360
                ******:**************:************:  **********************.

Ls1-FEH2        GKQLIQWPVEEIEALRQNEVKLEDTNLKPGSVLEIHGITAAQADVTISFKLENLKEAEVL    420
Ci1-FEH2a       GKQLIQWPVEEIEELRQNQVNLQNKNLKPGSVLEIHGIAASQADVTISFKLEGLKEAEVL    420
Ci1-FEH2b       GKQLIQWPIEEIEELRQNEVNLQNKNLKPGSVLEIHGITASQADVTISFKLEELKDAEVL    420
                ********:**** ****:*:*::.*************:*:*********** **:****

Ls1-FEH2        DTSSVDPQALCTERGASSEGAFGPFGLLAMASKDLEEQSAIFFRVFQNQNGRYSVLMCSD    480
Ci1-FEH2a       DTTLVDPQALCNERGASSRGALGPFGLLAMASKDLKEQSAIFFRVFQNQLGRYSVLMCSD    480
Ci1-FEH2b       DTTSVDPQVLCNERGASSRGAIGPFGLLAMASKDLKEQSAIFFRVFQNQLGRYSVLMCSD    480
                **: ****.**.******.**:*************:************* **********

Ls1-FEH2        LSRSTVRSNIDTTSYGAFVDIDPRSEEISLRNLIDHSIIESFGAGGKTCITSRIYPKFVK    540
Ci1-FEH2a       LSRSTVRSNIDTTSYGAFVDIDPRSEEISLRNLIDHSIIESFGAGGKTCITSRIYPKFVN    540
Ci1-FEH2b       LSRSTVRSNIDTTSYGAFVDIDPKSEEISLRNLIDHSIIESFGAGGKTCITSRIYPQFVN    540
                ***********************:********************************:**:

Ls1-FEH2        NEDAHLFAFNNGTQSVKISQMSAWSMKNAEFVVDQTVKSTM  581
Ci1-FEH2a       NEEAHLFVFNNGTQNVKISEMSAWSMKNAKFVVDQSVKSAA  581
Ci1-FEH2b       NEEAHLFAFNNGTQNVKISKMSAWSMKNAAFVVDQNVKSAA  581
                **:****.******.****:********* *****.***:  #

#  Percent Identity  Matrix - created by Clustal2.1
     1: Ls1-FEH2     100.00   90.19   89.33
     2: Ci1-FEH2a     90.19  100.00   94.84
     3: Ci1-FEH2b     89.33   94.84  100.00
```

Supplementary Figure S7: Amino acid sequence alignment and percent identity of 1-FEH2 proteins from chicory (Ci1-FEH2a, Ci1-FEH2b) and lettuce (Ls1-FEH2). Sequence alignment was performed using Clustal Omega (EMBL-EBI). Conserved residues are indicated with asterisks (*); strong and weak similarity are marked with colons (:) and periods (.), respectively.

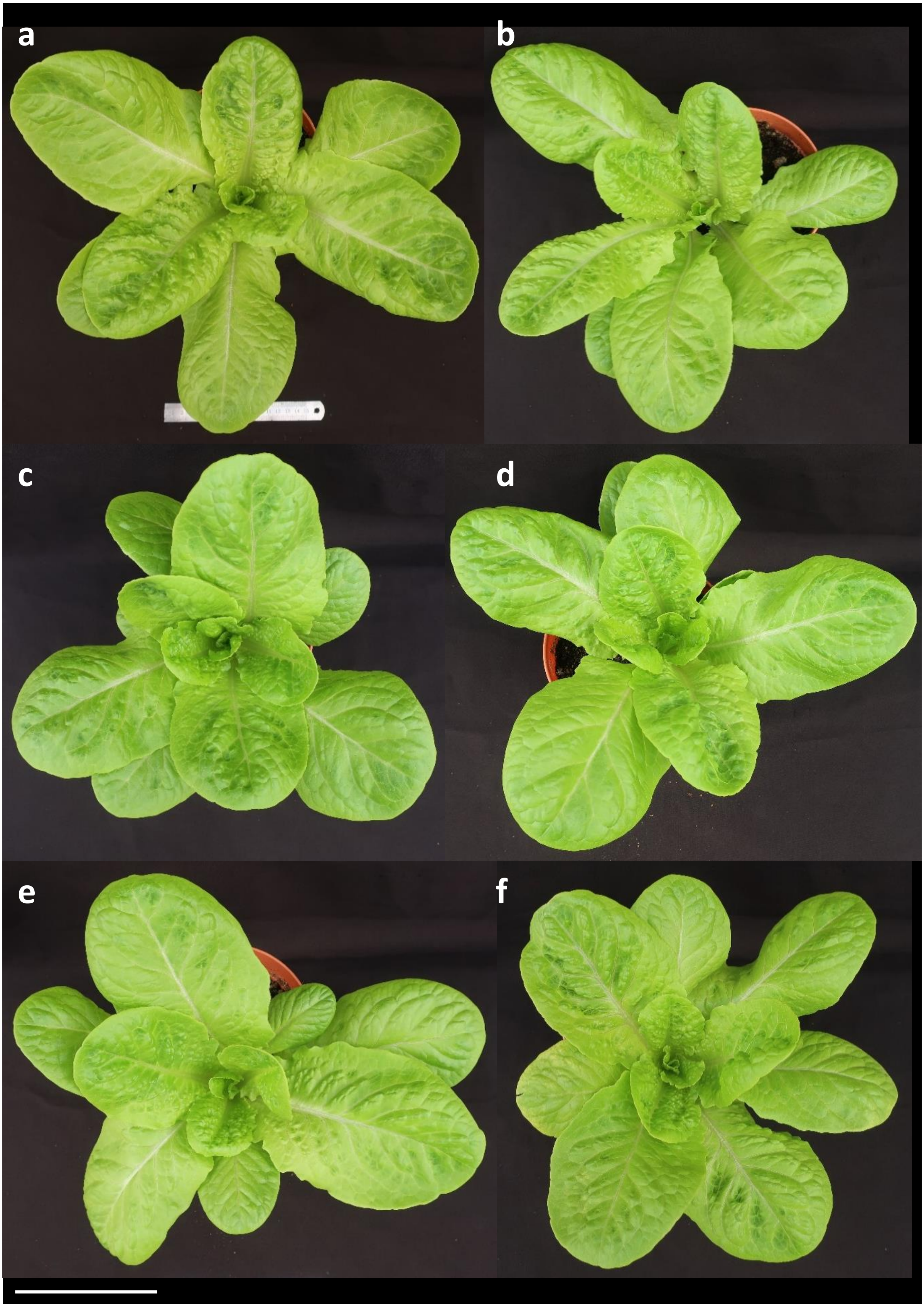

Supplementary Figure S8: Representative images of non-edited (NE) control and *1-FEH2* mutant plants grown under identical conditions. Bar (bottom left) = 15 cm. (a) Non-edited (NE) control. (b) H2A heterozygous mutant. (c) H2A homozygous mutant. (d) H2B homozygous mutant. (e) H2C homozygous mutant. (f) H2D homozygous mutant.

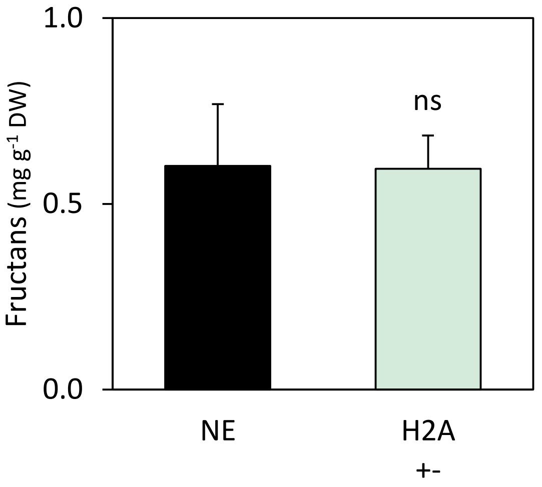

Supplementary Figure S9: Fructan content in two-month-old leaves of heterozygous *1-FEH2* mutant plants of line H2A (genotype +-) compared with non-edited (NE) control plants. Data represent means ± SEM (n = 3). No statistically significant difference in fructan content was found using one-way ANOVA. DW, dry weight.

a

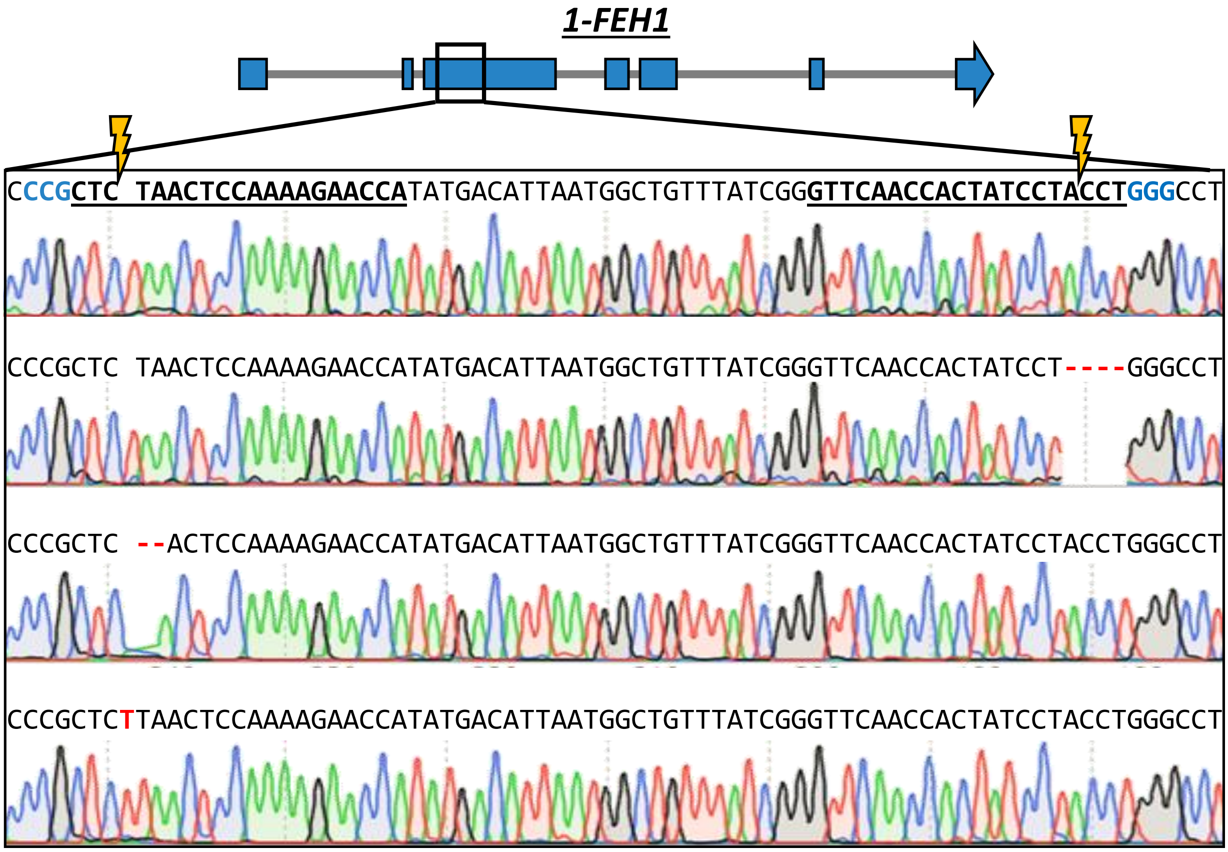

b

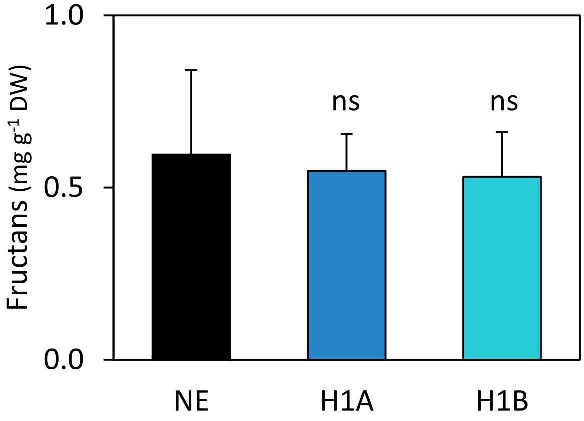

Supplementary Figure S10: Mutation sequences and fructan content in two-month-old independently generated *1-FEH1* gene-edited lettuce cv. 'Noga' plants. (a) *1-FEH1* gene structure and sequence in non-edited (NE) plants and three CRISPR-generated mutant lines (H1A, H1B and H1C). Two gRNA spacer sequences are underlined; PAM sequences are shown in blue. Base indels are indicated in red; the size of the indels for each mutant line is displayed to the left. (b) Fructan content in two-month-old leaves of heterozygous *1-FEH2* mutant plants (H2A, genotype +-) compared with non-edited (NE) control plants. Data represent means ± SEM (n = 3). No statistically significant difference in fructan content was found compared with the control using one-way ANOVA. DW, dry weight.

Supplementary Table S1: Regeneration efficiency of lettuce cv. 'Noga' cotyledons cultured on media with varying concentrations and ratios of cytokinin (6-benzylaminopurine, BA) and auxin (1-naphthaleneacetic acid, NAA) after 21 days. Regeneration efficiency is expressed as the percentage of viable regenerated plantlets relative to the total number of initial explants. The tested media included six combinations of BA and NAA, with cytokinin-to-auxin ratios ranging from 1:1 to 12.5:1.

Regeneration Medium	BA (mg/l)	NAA (mg/l)	Cytokinin/Auxin (ratio)	Regeneration efficiency
#1	0.1	0.1	1:1	26.4%
#2	0.05	0.05	1:1	22.7%
#3	0.1	0.05	2:1	11.1%
#4	0.2	0.05	4:1	8.1%
#5	0.5	0.1	5:1	71.5%
#6	0.5	0.04	12.5:1	87.3%

Supplementary Table S2: Primer list.

Name	Purpose	Sequence (5' --> 3')
1-FEH1_F	Sequencing	TGTCAATCTGGACCGCTTGC
1-FEH1_R	Sequencing	TCCATTTTCCATCTGGGCCG
1-FEH1_RT_F	qPCR	ATCACTGCATCACAGGCTGA
1-FEH1_RT_R	qPCR	ACCTCCCACTGATAGACGCA
1-FEH2 R	Sequencing	TGCGAGGTCTTGGACTTGAC
1-FEH2_F	Sequencing	GACAGCTTTGGTCCACGTTC
1-FEH2_RT_F	qPCR	GTGTAGCGATCTTAGCAGGTCT
1-FEH2_RT_R	qPCR	ACATGTCTTCCCACCAGCTC
1-FFT_RT_F	qPCR	TGACGAGGCACGTTCAGAAT
1-FFT_RT_R	qPCR	ACCGAATGATCCACCAGTAACC
1-SST_RT_F	qPCR	CTACGGCGCTGCTAAGCTAT
1-SST_RT_R	qPCR	CAAGGAGGGACTTCAACCA
Actin_F	qPCR	AGTCTGGCCCATCCATTGTT
Actin_R	qPCR	TCATCCCCAGTCCCATACCC
CCD4a_F	Sequencing	TGCAAGTTTTGGAATACAGCTCA
CCD4a_R	Sequencing	GGACCGCGTGGTAGATACTG
CSN5_F	Sequencing	CGTAGAAACGACGTGACATCCA
CSN5_R	Sequencing	CTCATAGGCATCGGCCTGAG
GGP2_F	Sequencing	CCCTCTTCCCCTTTGCTGATT
GGP2_R	Sequencing	CCCCTGTATTCAACCCCAGG
hCas9_F	Sequencing	TGGAGGAGTCCTTTTTGGTG
hCas9_R	Sequencing	GCTTTGGTGATCTCCGTGTT
HMG1_F	Sequencing	AGACGTAAACGACAACTCCGA
HMG1_R	Sequencing	TCGACTTCACCTTCAAGCACA
LCYe-F	Sequencing	GCTAAAAGCGACCGTTGTGT
LCYe-R	Sequencing	CACAGACAGACTTTTAGTGTCCC
pTRV2_DsRed_F	PCR for viral genes	GTTTTAATTAACTTCTCCTACGCA
pTRV2_DsRed_R	PCR for viral genes	GTATCACCCACCCTCTGAATTTC
TIP41_F	qPCR	TTTGTATGGAGATGAATTGGCTGATA
TIP41_R	qPCR	CGTAAGAGAAGAAACCAACAGCTAGG
